# GLP-1 receptor agonist therapy and pregnancy: Evolving and emerging evidence

**DOI:** 10.1016/j.clinme.2025.100298

**Published:** 2025-02-22

**Authors:** Maria S Varughese, Fidelma O'Mahony, Lakshminarayanan Varadhan

**Affiliations:** a University of Sheffield Medical School, Royal Hallamshire Hospital, Sheffield S10 2RX, South Yorkshire, UK; b Department of Obstetrics & Gynaecology, University Hospitals of North Midlands NHS Trust, Stoke-on-Trent, UK; c School of Medicine, Faculty of Health, Keele University, North Staffordshire, UK; d Department of Diabetes & Endocrinology, University Hospitals of North Midlands NHS Trust, Stoke-on-Trent, UK

**Keywords:** GLP-1 receptor agonist therapy, Pregnancy, Type 2 diabetes mellitus, Fetal medicine

## Abstract

The prevalence of type 2 diabetes mellitus (T2DM) and obesity is increasing in young adults, posing significant risks around pregnancy. Obesity also impacts on fertility and the co-existence of polycystic ovarian syndrome increases the prevalence of cardiovascular metabolic risk factors. There has been a renewed interest in glucagon-like peptide-1 receptor agonists (GLP-1RA) in this context, due to their multi-dimensional impact on the reproductive axis, as well as their ability to simultaneously target weight loss and glycaemic control. There is, however, limited availability of safety data with respect to these newer non-insulin-based diabetes medications from the perspective of fetal development. As GLP-1RA are not licensed for use in pregnancy, with the increasing chances of incidental exposure from pre-conception use for obesity and T2DM, it is imperative that pre-conception counselling should be an integral part of consultation prior to the initiation of these drugs.

The prevalence of type 2 diabetes mellitus (T2DM) and obesity in pregnant women is increasing, aligned with the global epidemic.^1^ This warrants an ever-increasing need to treat hyperglycaemia, taking into account the implications of the medications around the time of conception.^2^ Conventionally, insulin therapy has been the favoured treatment option, with only limited evidence of safety data available on non-insulin-based diabetes medications from the perspective of fetal development.^3^ There is an increasing trend of intentionally or unintentionally using non-insulin-based treatment for managing T2DM during pregnancy, with a progressive interest towards glucagon-like peptide-1 receptor agonists (GLP-1RA) in this context.^4^

## Pre-pregnancy effectiveness

The use of GLP-1RA in a pre-pregnancy setting is relevant in the context of managing obesity, polycystic ovarian syndrome (PCOS) or control of hyperglycaemia in T2DM. The distribution of GLP-1 receptors (GLP-1R) from an anatomical aspect throughout the reproductive system, and the observed effects in pre-clinical models as well as clinical studies, indicate that they could be a central modulator of signals connecting the reproductive and the metabolic systems, thereby having a multi-dimensional impact.^4^ GLP-1RA could exert anti-inflammatory effects on various peripheral reproductive organs too, which is a central pathogenetic mechanism in all of these conditions.^5^, ^6^, ^7^

PCOS is a leading cause for infertility, being the most common endocrine disorder in women of the reproductive age group.^5^ Though the diagnostic criteria for PCOS include hyperandrogenism, oligo-anovulation, and polycystic ovary morphology, central adiposity has got a convincing impact across different PCOS phenotypes, affecting the management of symptoms and outcomes of fertility.^5^^,^^6^ This also contributes to hyperinsulinaemia, thereby increasing the risk of metabolic syndrome, characterised by glucose intolerance, insulin resistance, dyslipidaemia and hypertension.^6^^,^^7^ The co-existence of metabolic syndrome among women with PCOS is thought to range between 33% and 46%.^5^ In addition to the undesirable consequences of obesity and insulin resistance on female fertility, metabolic disorders can directly or indirectly also impact on the fertility of women by affecting either the pituitary–hypothalamic function or ovarian function.^5^^,^^6^ GLP-1RA provides the benefit of improving metabolic parameters, facilitating weight loss and enhancing insulin sensitivity.^5^^,^^6^ The use of GLP-1RA has been shown to enhance menstrual regularity, boost ovulation, and increase pregnancy rates in women with obesity and PCOS.^6^^,^^7^ These drugs can also positively alter hormonal parameters, including decreasing free androgen levels and increasing sex hormone binding globulin (SHBG) levels, which are of further benefit.^7^

Obesity per se accounts for the increased risk of infertility, mainly linked to dysfunction in the hypothalamic–pituitary ovarian (HPO) axis, impaired oocyte quality and alterations in endometrial receptivity.^5^ This also contributes to poor reproductive outcomes, irrespective of the mode of conception.^6^ Fortunately, a substantial reduction in body weight has been demonstrated to improve not only various metabolic parameters such as hyperlipidaemia, glycaemic control and hypertension, but also hyperandrogenism and reproductive function in women with PCOS.^7^^,^^8^ The use of GLP-1RA is associated with significant weight loss in patients without T2DM, which could therefore enhance the improvement in metabolic factors too.^9^ With the use of GLP-1RA being licensed for weight loss alone in some countries, the exposure risk is a valid concern for planning pregnancy.

The risks of hyperglycaemia and sub-optimal metabolic control in pregnancy are well established. Obesity is quite prevalent among people with T2DM who become pregnant (65%) and glycaemic control and high BMI are individual risk factors for unfavourable pregnancy outcomes, and far from ideal HbA1c levels in the first trimester are associated with congenital anomalies.^10^ Treatment with GLP-1RA therefore offers the opportunity to simultaneously target hyperglycaemia, excess body weight with associated metabolic factors, while also improving fertility.^11^ Murphy *et al* demonstrated the unintentional use of statins and angiotensin converting enzyme inhibitors to be as high as 5%.^10^ With approximately 50% of pregnancies seen in clinical practice being unplanned and lower availability of pre-conception clinics, the chance of incidental exposure to GLP-1RA is likely to increase.

A comprehensive, multidisciplinary approach for weight management, both in those planning to conceive naturally or following any method of assisted reproductive technology, should therefore be an integral part of pre-conception clinics and during consultations at the point of initiation of GLP-1RA.^5^, ^6^, ^7^, ^8^

### Antenatal implications

With increasing use of GLP-1RA for obesity and T2DM, the risk of inadvertent exposure of these drugs during the first trimester is likely to increase. Being quite large molecules, theoretically it may be less likely to cross the placenta early in pregnancy to cause any deleterious effects to the fetus. A systematic review of existing evidence on GLP-1RA during pregnancy in animal studies demonstrated that exposure of mice to GLP-1RA was associated with (a) decreased fetal weight and growth, (b) delayed ossification, (c) skeletal variants, and (d) visceral abnormalities.^11^

A recently published cohort study by the InPreSS consortium included more than 50,000 pregnancies in women with pregestational T2DM from six different countries. It demonstrated the increase in peri-conceptional use of second-line non-insulin-based treatment, especially of GLP-1RA in the USA, highlighting the general trend of the shift in modality of treating T2DM in the reproductive age group globally. There was no increased risk of major congenital malformations (MCMs) after peri-conceptional exposure to GLP-1RA, or any of the other second-line non-insulin-based class of medications in this study.^12^

A further prospective multicentre observational study by Dao *et al* assessed pregnancy outcomes following exposure to GLP-1RA in early pregnancy.^13^ This study evaluated pregnant women exposed to GLP-1RA during the first trimester of pregnancy [n=168] with reference to two other comparator cohorts of pregnant women (one group with T2DM [n=156] and the other group being overweight or obese pregnant women [n=163] without T2DM). The study did not recognise a specific pattern of birth defects; MCMs when compared with diabetes [2.6% vs 2.3%; adjusted OR, 0.98 (95% CI, 0.16–5.82)] or to overweight and obese [2.6% vs 3.9%; adjusted OR 0.54 (0.11–2.75)]. Cumulative incidences for live births, pregnancy losses and pregnancy terminations were 59%, 23% and 18% compared to 69%, 26% and 6% in the diabetes group and 63%, 29% and 8% in the obesity group respectively. In comparison with insulin treatment, neither of these prospective studies found any increased risk of MCMs for infants exposed to GLP-1RA inadvertently during the very crucial peri-conceptional period.^12^^,^^13^

Even though these studies do not suggest that these medications have strong teratogenic effects, further research is warranted to fully assess the safety of these medications in pregnancy. Observational studies may demonstrate evidence of safety of incidental exposure of GLP-1RA during the first trimester. Until prospective trials and safety data are available, it is important to balance the risk of continuing GLP-1RA until confirmation of pregnancy vs. prematurely stopping these medications and risking reversal of the achieved glycaemic control and benefits of weight loss attained.^11^, ^12^, ^13^ Furthermore, GLP-1RA with longer half-life such as semaglutide may theoretically risk longer exposure in comparison to shorter-acting drugs such as liraglutide despite stopping treatment as soon as pregnancy is confirmed. It is therefore pertinent that a fully informed discussion, including contraceptive advice, be conducted with patients treated with GLP-1RA during consultation in the pre-conception clinics.

### Postnatal perspective

There are fewer than 10 case reports published in the literature of congenital anomalies with inadvertent exposure to GLP-1RA during pregnancy (exenatide, liraglutide, semaglutide and dulaglutide), to the best of our knowledge,^14^, ^15^, ^16^, ^17^, ^18^, ^19^, ^20^, ^21^, ^22^ In all the case reports published to date, GLP-1RA was stopped as soon as they were found to be pregnant, except in one woman where treatment was continued until the end of pregnancy to sustain optimal glycaemic control. The exposure in these cases had variable timelines across the trimesters (from pre-conception to 4 weeks or more through the first trimester and even after 30 weeks to full-term) before they were stopped.

One baby had mild bilateral renal pyelectasis with no other congenital anomalies or maternal complications.^20^ In one woman who had been exposed to liraglutide from pre-conception to 17 weeks, her infant had been diagnosed with an atrial septal defect that corrected spontaneously by age 3 years.^22^ She had a subsequent pregnancy again with exposure to liraglutide for a similar duration, with no fetal or maternal complications. Neonatal hypoglycaemia was reported in one woman,^16^ and significant weight gain of 35 kg in another woman leading to macrosomia with shoulder dystocia.^18^ Polyhydramnios was reported, in a patient who presented very late at 33 weeks of gestation and in her sixth pregnancy, but no MCMs or fetal abnormalities were detected.^21^

The use of GLP-1RA during lactation is another contentious situation. As GLP-1 is a large protein, its excretion is likely to be significantly low in breast milk, and the secreted molecule is likely to be digested by the fetal gastrointestinal system.^12^

### Metformin simile

In this context, it is also worth remembering that the use of metformin in pregnancy was not adopted by randomised control trials, but based on safety and efficacy experience with the drug and the priority not to derange glycaemic control during pregnancy by withdrawing this treatment. Several studies subsequently have found no convincing detrimental consequences with metformin use during preganancy.^24^, ^25^, ^26^, ^27^

### GLP-1RA and male fertility

Likewise, the impact of GLP-1RA treatment on male fertility is also relevant here given the topical theme. Experimental and clinical studies have explored this aspect and other than the beneficial effects on fertility in men with the weight loss achieved, no harmful side effects have been noticed on fetal outcomes from observational data available to date.^28^, ^29^, ^30^, ^31^

## Conclusion

The prevalence of people who could be overweight or obese, with or without T2DM, continues to rise in the general population, and there will be further widening of the landscape of indications for treatment with GLP-1RA. In the fullness of time, it will be prudent to be able to educate and counsel patients in a comprehensive manner about treatment with GLP-1RA, especially with the newer dual incretin mimetic twincretin, tirzepatide that is already widely available and the feasibility of novel next-generation triple-hormone-receptor agonist, retatrutide on the horizon.

As things stand, there is not enough evidence to support safe use of GLP-1RA during pregnancy or to effectively counsel patients about peri-conceptional exposure. Patients taking GLP-1RA should be advised to use contraception to avoid inadvertent exposure early in pregnancy. Furthermore, pre-conception counselling regarding management of hyperglycaemia and adequate drug-free interval from GLP-1RA treatment as per manufacturer's recommendation should be considered before women with T2DM contemplate pregnancy. We still require additional data about the risks regarding the potential effects on (a) fetal development, (b) teratogenicity, (c) spontaneous abortion, (d) fetal growth, (e) maternal glycaemic control, (f) pre-term birth and (g) placental and breast milk drug passage.^23^

Interestingly, a very recent study reported that the prescription usage of GLP-1RA within 24 months preceding a pregnancy was associated with a reduced risk of several adverse obstetric outcomes, including gestational diabetes mellitus, hypertensive disorders of pregnancy, preterm delivery and caesarean delivery.^32^ These data suggest that the use of GLP-1RA may be a powerful tool to improve perinatal outcomes in high-risk populations. Further research is most certainly indicated to navigate and define how GLP-1RA is best incorporated clinically into pre-conception health optimisation strategies to benefit the wider population, especially with newer combination agents in the horizon.

## Funding

This article did not receive any specific grant from funding agencies in the public, commercial, or not-for-profit sectors.

## CRediT authorship contribution statement

**Maria S Varughese:** Writing – review & editing, Writing – original draft, Formal analysis, Data curation, Conceptualization. **Fidelma O'Mahony:** Writing – review & editing, Formal analysis, Data curation. **Lakshminarayanan Varadhan:** Writing – review & editing, Writing – original draft, Validation, Supervision, Formal analysis, Data curation, Conceptualization.

## Declaration of competing interest

The authors declare that they have no known competing financial interests or personal relationships that could have appeared to influence the work reported in this paper.
